# Ressonância Magnética Cardíaca e Histologia na Valvopatia Aórtica: Convergências e Limitações

**DOI:** 10.36660/abc.20260140

**Published:** 2026-04-23

**Authors:** Helder Jorge Andrade Gomes, Tiago Senra

**Affiliations:** 1 Faculdade de Medicina de Jundiaí Jundiaí SP Brasil Faculdade de Medicina de Jundiaí, Jundiaí, SP – Brasil; 2 Vera Cruz Hospital Campinas SP Brasil Vera Cruz Hospital, Campinas, SP – Brasil; 3 ICON Diagnósticos por Imagem Jundiaí SP Brasil ICON Diagnósticos por Imagem, Jundiaí, SP – Brasil; 4 Instituto Dante Pazzanese de Cardiologia São Paulo SP Brasil Instituto Dante Pazzanese de Cardiologia, São Paulo, SP – Brasil

**Keywords:** Imageamento por Ressonância Magnética, Fibrosis, Valvopatia Aórtica

A fibrose miocárdica constitui elemento central do remodelamento ventricular na valvopatia aórtica grave e associa-se à disfunção ventricular e a piores desfechos, inclusive após intervenção valvar.^1-3^ Embora a fração do volume de colágeno obtida por histologia represente o padrão-ouro para quantificação da fibrose intersticial, a biópsia miocárdica não integra a prática clínica rotineira devido ao seu caráter invasivo e às limitações amostrais. Nesse contexto, a ressonância magnética cardíaca (RMC) se consolidou como método não invasivo para caracterização tecidual, permitindo identificar fibrose regional por meio do realce tardio e estimar expansão intersticial por meio do mapeamento T1 e do cálculo do volume extracelular (VEC).^4-7^

O estudo publicado nesta edição dos Arquivos Brasileiros de Cardiologia contribui de forma relevante ao comparar diretamente parâmetros de RMC com a fração do volume de colágeno obtida por biópsia miocárdica em 108 pacientes com valvopatia aórtica grave submetidos à cirurgia.^8^ Diferentemente de muitas séries anteriores compostas exclusivamente por estenose aórtica, a coorte incluiu também pacientes com insuficiência aórtica, ampliando a aplicabilidade dos achados e incorporando um espectro fisiopatológico mais abrangente de sobrecarga ventricular.

Os autores demonstram que tanto o realce tardio quanto o VEC global médio apresentaram correlação moderada com a fibrose histológica no septo interventricular. O realce tardio exibiu melhor desempenho diagnóstico global, com maior acurácia e área sob a curva ROC superior à do VEC. Este último apresentou sensibilidade limitada para identificação de maior carga fibrótica definida pela mediana da fração de colágeno, além de baixa concordância com a histologia.

Esses achados reforçam que os métodos não são equivalentes e provavelmente captam dimensões distintas do processo fibrótico. O realce tardio depende da diferença relativa de sinal entre miocárdio normal e áreas com maior acúmulo de colágeno, sendo mais sensível à fibrose focal ou substitutiva. Já o VEC reflete a expansão do compartimento extracelular, que pode decorrer não apenas da deposição de colágeno, mas também de inflamação, remodelamento intersticial heterogêneo ou infiltração, como ocorre na amiloidose – condição que pode coexistir em pacientes idosos valvopatas graves.^9^ Ainda que o VEC sofra menor influência de diversos fatores do que o valor de T1 nativo, como idade, sexo, frequência cardíaca, sequência de pulso utilizada, força e homogeneidade do campo magnético do aparelho de ressonância, cabe ressaltar que ainda não há na literatura consenso sobre qual seria o valor normal de VEC miocárdico. Assim, a correlação apenas moderada entre imagem e histologia provavelmente traduz a complexidade biológica do fenômeno, e não necessariamente limitação técnica isolada.

Outro aspecto relevante diz respeito à distribuição espacial da fibrose. A fibrose na valvopatia aórtica apresenta distribuição espacial variável e não restrito ao septo interventricular.^2,10^ A comparação escolhida pelos autores entre método global de avaliação da fibrose miocárdica e amostras histológicas focais envolve limitações inerentes de representatividade. Ainda assim, o estudo demonstra que a RMC é capaz de identificar de forma consistente a presença de remodelamento fibrótico relevante, mesmo que não reproduza com precisão absoluta a quantificação histológica.

Do ponto de vista clínico, o realce tardio já demonstrou associação prognóstica consistente em pacientes com estenose aórtica e permanece marcador consolidado de fibrose estabelecida.^2,3^ O VEC, por sua vez, tem se mostrado associado ao prognóstico em pacientes com estenose aórtica moderada ou importante, inclusive assintomáticos, sugerindo potencial papel na estratificação mais precoce.^11^ Além disso, evidências recentes indicam que o VEC apresenta redução após a intervenção cirúrgica ou percutânea na estenose aórtica, o que abre perspectiva para sua utilização na definição do melhor momento para intervenção.^12^

Entretanto, a integração entre realce tardio e VEC pode oferecer visão mais abrangente do processo fibrótico na doença valvar, captando tanto componentes regionais quanto intersticiais, mas ainda não integra o estadiamento proposto por sociedades internacionais – particularmente quanto ao momento ideal de intervenção – campo que permanece em evolução.

Ao correlacionar histologia e RMC em uma coorte cirúrgica que incluiu tanto estenose quanto insuficiência aórtica, o estudo amplia a compreensão sobre convergências e limites entre métodos invasivos e não invasivos na avaliação da fibrose miocárdica. Seus achados reforçam que a fibrose na valvopatia aórtica é fenômeno multifacetado e que sua caracterização por imagem deve ser interpretada de forma contextualizada. A consolidação do papel prognóstico desses marcadores e sua eventual incorporação na tomada de decisão terapêutica representam o próximo desafio na abordagem da valvopatia aórtica.
