# Supplementary material for: Ressonância Magnética Cardíaca e Histologia na Valvopatia Aórtica: Convergências e Limitações
Source: Arq Bras Cardiol. 2026 Apr 23;123(2):e20260140. [Article in Portuguese] doi: 10.36660/abc.20260140 (PMC13128185; doi:10.36660/abc.20260140)
Supplement: Translation

# Translation: Cardiac Magnetic Resonance Imaging and Histology in Aortic Valve Disease: Convergences and Limitations

**DOI:** 10.36660/abc.20260140i

**Authors:** Helder Jorge Andrade Gomes, Tiago Senra

**Affiliations:** 1 Jundiaí SP Brazil Faculdade de Medicina de Jundiaí, Jundiaí, SP – Brazil; 2 Campinas SP Brazil Vera Cruz Hospital, Campinas, SP – Brazil; 3 Jundiaí SP Brazil ICON Diagnósticos por Imagem, Jundiaí, SP – Brazil; 4 São Paulo SP Brazil Instituto Dante Pazzanese de Cardiologia, São Paulo, SP – Brazil

**Keywords:** Magnetic Resonance Imaging, Fibrosis, Aortic Valve Disease

Myocardial fibrosis is a central element of ventricular remodeling in severe aortic valve disease and is associated with ventricular dysfunction and worse outcomes, even after valve intervention.^1-3^ Although the collagen volume fraction obtained by histology represents the gold standard for quantifying interstitial fibrosis, myocardial biopsy is not part of routine clinical practice due to its invasive nature and sample limitations. In this context, cardiac magnetic resonance imaging (CMR) has become established as a non-invasive method for tissue characterization, allowing the identification of regional fibrosis through late enhancement and the estimation of interstitial expansion through T1 mapping and calculation of extracellular volume (ECV).^4-7^

The study published in this issue of the *Arquivos Brasileiros de Cardiologia* makes a significant contribution by directly comparing CMR parameters with the collagen volume fraction obtained by myocardial biopsy in 108 patients with severe aortic valve disease who underwent surgery.^8^ Unlike many previous series composed exclusively of aortic stenosis, this cohort also included patients with aortic insufficiency, broadening the applicability of the findings and incorporating a more comprehensive pathophysiological spectrum of ventricular overload.

The authors demonstrate that both late gadolinium enhancement and mean global ECV showed a moderate correlation with histological fibrosis in the interventricular septum. Late gadolinium enhancement exhibited better overall diagnostic performance, with greater accuracy and an area under the ROC curve superior to that of ECV. The latter showed limited sensitivity for identifying higher fibrotic burden defined by the median collagen fraction, in addition to low concordance with histology.

These findings reinforce that the methods are not equivalent and likely capture distinct dimensions of the fibrotic process. Late enhancement depends on the relative signal difference between normal myocardium and areas with greater collagen accumulation, being more sensitive to focal or replacement fibrosis. ECV reflects the expansion of the extracellular compartment, which can result not only from collagen deposition but also from inflammation, heterogeneous interstitial remodeling, or infiltration, as occurs in amyloidosis – a condition that can coexist in elderly patients with severe valvular heart disease.^9^ Although ECV is less influenced by several factors than the native T1 value, such as age, sex, heart rate, pulse sequence used, strength, and homogeneity of the magnetic field of the MRI machine, it should be noted that there is still no consensus in the literature on what the normal value of myocardial ECV would be. Thus, the only moderate correlation between imaging and histology likely reflects the biological complexity of the phenomenon, and not necessarily an isolated technical limitation.

Another relevant aspect concerns the spatial distribution of fibrosis. Fibrosis in aortic valve disease presents a variable spatial distribution and is not restricted to the interventricular septum.^2,10^ The comparison chosen by the authors between a global method of assessing myocardial fibrosis and focal histological samples involves inherent limitations of representativeness. Even so, the study demonstrates that CMR can consistently identify the presence of relevant fibrotic remodeling, even if it does not reproduce the histological quantification with absolute precision.

From a clinical standpoint, late gadolinium enhancement has already demonstrated a consistent prognostic association in patients with aortic stenosis and remains a consolidated marker of established fibrosis.^2,3^ The ECV, in turn, has been shown to be associated with prognosis in patients with moderate or severe aortic stenosis, including asymptomatic patients, suggesting a potential role in earlier stratification.^11^ Furthermore, recent evidence indicates that ECV decreases after surgical or percutaneous intervention in aortic stenosis, which opens up perspectives for its use in defining the best time for intervention. ^12^

However, the integration between late gadolinium enhancement and ECV can offer a more comprehensive view of the fibrotic process in valvular heart disease, capturing both regional and interstitial components, but it does not yet integrate the staging proposed by international societies – particularly regarding the ideal timing of intervention – a field that remains under evolution.

By correlating histology and CMR in a surgical cohort that included both aortic stenosis and aortic insufficiency, the study broadens the understanding of convergences and limitations between invasive and non-invasive methods in the evaluation of myocardial fibrosis. Its findings reinforce that fibrosis in aortic valve disease is a multifaceted phenomenon and that its characterization by imaging should be interpreted contextually. The consolidation of the prognostic role of these markers and their eventual incorporation into therapeutic decision-making represent the next challenge in the approach to aortic valve disease.
